# Clenched fist syndrome: a case report

**DOI:** 10.1186/s13256-018-1729-5

**Published:** 2018-06-18

**Authors:** Trygve Nissen, Rolf Wynn

**Affiliations:** 1 Division of Mental Health and Addictions, University Hospital of North Norway, N-9291 Tromsø, Norway; 2 Department of Clinical Medicine, Faculty of Health Sciences, UiT – The Arctic University of Norway, N-9037 Tromsø, Norway

**Keywords:** Clenched fist syndrome, Psycho-flexed hand, Conversion disorder, Factitious disorder

## Abstract

**Background:**

The clenched fist syndrome/psycho-flexed hand, first described in the early 1980s, has not yet entered the major psychiatric textbooks. Curiously, the phenomenon has been illuminated mainly in journals and textbooks on hand surgery. There is a need to examine, describe, and understand this syndrome from a psychiatric perspective.

**Case presentation:**

We present a case we encountered in an intensive care unit. A 60-year-old white man with schizophrenia, cerebrovascular disease, diabetes mellitus type 2, and peripheral neuropathy, developed rather acutely bilateral clenched fists in the aftermath of a traumatic dislocated hip fracture that was operated on. He later died due to complications from the surgical procedure.

**Conclusions:**

While this was a complex case with some clinical uncertainty regarding the cause of our patient’s symptoms, we conclude that psychological processes were central to the development of his clenched fists. The phenomenon of clenched fists and our case are discussed with reference to the accumulated literature on psychogenic hand disorders and the *International Statistical Classification of Diseases and Related Health Problems*, 10th version. The nosological status appears to be obscure. This case presentation is a first step in increasing the understanding of this syndrome from a psychiatric perspective.

## Background

Medical disorders mimic psychiatric ones, and vice versa. In an often quoted article, the Canadian psychiatrist Erwin K. Koranyi wrote: “No single psychiatric symptom exists that cannot at times be caused or aggravated by various physical illnesses” [1]. The causality can go in the opposite direction as well. Often physical symptoms and signs are caused or aggravated by psychological factors or psychiatric illness. And sometimes the psychiatric symptoms are less prominent than the physical signs. In conversion or dissociative disorders, patients can have various physical signs, for example paralysis or anesthesia, and still be unconcerned about their impairment, that is, feel no or only minor subjective suffering. This phenomenon or sign of calm acceptance has been called “*la belle indifférence*.” Its presence can be helpful in the diagnostic work-up although some researchers have disputed its assumed high prevalence in conversion disorders [2].

We encountered a patient with clenched hands that challenged our diagnostic skills. As the literature on the subject is parsimonious, we feel obliged to report our case to the medical community. Clinical case reports can be very useful in detecting rare disorders and generating hypotheses and may give an in-depth understanding of significant educational value. The case report genre appears to be the most appropriate method for conveying our observations [3–5]. The case report follows the CARE guidelines [6].

## Case presentation

A 60-year-old white unmarried man with chronic schizophrenia fell to the floor and was unable to get up or walk. When examined he had an asymmetrical smile and apparent paresis of his left leg. He was hospitalized with a tentative diagnosis of stroke.

### Past history

From his relatives we learned that he had grown up in a village on the Norwegian coastline as the fourth of five siblings. He did not excel at school, and started at an early age to work in the local fishing industry. He held the job until at the age of 30 he moved to another part of the country. There he worked as a custodian at a hotel. At age 37 he went back to his home village to live close to his compassionate family of origin. He was then employed as an assistant custodian (supported employment) in the local fishing industry until he was 56-years old. He was treated for psychotic symptoms on-and-off from his mid-twenties. He was not diagnosed as having schizophrenia until he was 40-years old. Since then he received out-patient psychiatric treatment until the present illness occurred. At the age of 55, diabetes mellitus type 2 was diagnosed. Osteoporosis was diagnosed 2–3 months prior to the present illness.

### Present illness

This was the first time he had been hospitalized. His family members said that he had had swallowing problems, difficulties with speech, and unsteady gait for the last 4–5 years. This information was corroborated by our patient’s general practitioner. He had deteriorated physically over the last 3–4 months with increased fatigue. He had developed general inertia and was easily exhausted after a short period of physical labor. He had developed hypersomnia, with 10–12 hours of sleep per night, a weight loss of 4–5 kg, and an unsteady gait. To descend the stairs he preferred to sit on his buttocks and slide down the staircase one step at a time until reaching the lower floor. He had been a heavy tobacco smoker for several decades. His alcohol use was modest.

His main psychiatric symptoms before being hospitalized were social withdrawal and delusions about several small persons, the size of dolls, attached to his body. Furthermore, he had auditory and visual hallucinations. He was very reluctant to talk about the contents of the, probably imperative, auditory hallucinations. Antipsychotic medication, risperidone tablets, was first started in 1997. A year later the medication was switched to olanzapine tablets. The dosage varied between 7.5 and 15 mg per day without any objective or subjective side effects. There had been no unambiguous extrapyramidal side effects.

On physical examination, he was alert and orientated, but in some pain in his left hip and knee. He was afebrile with a body temperature (ear) of 37.4 °C, blood pressure was 136/83 mmHg, he had a regular pulse rate at 82 per minute, and oxygen saturation (SaO_2_) was 97%. Auscultation of his carotid arteries revealed no bruits. His heart rhythm was regular without any pulse deficit. There were no heart murmurs. A lung examination was suboptimal as inspiration was weak. It was possible that some crackles could be heard bilaterally at the base of his lungs. A neurological examination revealed impaired tongue wiggling when tested for quick side-to-side movements, dysarthria, symmetrically reduced muscle force (5−/4+) in his upper extremities, reduced force in his left leg (not quantified), and asymmetrical plantar reflexes (downward movement on the right side, indifferent on the left). His regular medication before admission was olanzapine tablets 12.5 mg/day (7.5 mg + 5 mg), metformin tablets 500 mg three times a day, calcium/cholecalciferol 500 mg/400 IU tablets two times a day, and paracetamol 500 mg two times a day. His complete blood count was normal: hemoglobin (Hgb) was 14.0 g/dL, hematocrit was 0.44, his white blood cell count was 8.2 × 10^9^/L, his platelet count was 275 × 10^9^/L, his neutrophil count was 5.6 × 10^9^/L, his lymphocyte count was 1.5 × 10^9^/L, his monocyte count was 0.7 × 10^9^/L, his eosinophil count was 0.4 × 10^9^/L, and his basophil count was < 0.1 × 10^9^/L. The only pathological tests from the chemistry panel were a low creatinine level of 59 μmol/L (reference range, 60–105), a high glucose level of 10.0 mmol/L (reference range, 4.0–6.0), a high glycated hemoglobin (HbA1c) level of 7.5% (reference range 4.3–6.1), a high alanine transaminase level of 94 U/L (reference range 10–70), and a high alkaline phosphatase level of 130 U/L (reference range 35–105). Urine, collected from a urine catheter on the day of admission, was delivered immediately to the microbiology laboratory in the same hospital building, and cultivated. There were > 100,000 bacteria per ml, identified as *Staphylococcus epidermidis*, probably representing contamination. There was no bacterial growth in a repeat urine test taken 3 days later.

The day after admission a left dislocated hip fracture was identified. This information, in combination with a normal cerebral magnetic resonance imaging (MRI) and disappearance of his facial asymmetry, caused the clinicians to reject the stroke hypothesis. His hip fracture was operated on the following day. Antibiotic medication (cefalotin 2 grams administered intravenously) was given twice: at the beginning and at the end of the surgery. Blood cultures with two sets, each consisting of one aerobic and one anaerobic bottle (Virtuo® blood culture, bioMérieux), were taken from his antecubital veins the day after admission. The cultures were brought to the microbiology laboratory immediately for further cultivation. No bacterial growth was seen.

Although the hip surgery was technically successful, it was not possible to physically mobilize our patient. The 12th day after hospital admission, a psychiatrist was consulted as our patient suffered from clouding of consciousness, episodic agitation, and increased anxiety. Olanzapine tablets were increased from 12.5 mg to 15 mg per day. On day 15 he was transferred to an acute psychiatric ward as it was considered the appropriate place for further treatment. This was unsuccessful as he deteriorated physically. As a consequence, he was returned to the intensive care unit. He was diagnosed as having bilateral lung emboli and suspected sepsis. New blood cultures were taken. Cefotaxime administered intravenously, 1 g three times a day, was started on day 18. Two days later, the cefotaxime dosage was increased to 2 g three times a day. The blood cultures revealed no growth.

Unfortunately, from now on a clinical downhill course followed. Our patient got aspiration pneumonia and was unable to swallow food or fluids. It was decided to stop further oral nutrition (fluids, food, pills) in an attempt to prevent further aspirations to his lungs. Instead, total parenteral nutrition was started. The tentative neurological diagnoses being discussed at this point were motor neuron disease, diabetic neuropathy, and extrapyramidal side effects of antipsychotics.

On the 20th day, a neurological examination found only slightly reduced muscle strength (grade 4–4+) for adduction and abduction of his shoulders bilaterally and a tendency to lead pipe rigidity in his wrist joints. No conclusive diagnosis was made. Three days later (day 23), a repeat neurological examination by another neurologist showed essentially the same clinical picture. The lead pipe rigidity in his upper extremities lessened significantly, almost to normal muscle tone, when our patient managed to relax. However, his wrist joints were strongly flexed and his hands tightly clenched to the bed rails bilaterally. Still, no conclusive neurological diagnosis was made. A videofluoroscopic swallow study and an assessment by a speech therapist were suggested but never performed because he did not regain the ability to cooperate.

The 23rd day was also the time for the second psychiatric consultation at the intensive care unit. Our patient was awake with a clear consciousness. He was oriented for time, place, and situation. Rapport was satisfactory. He was relaxed when engaged in a conversation or otherwise taken care of in his room; when left alone, he was stressed and obviously not at ease. He denied hallucinations. However, his dysarthric speech was a hindrance to an adequate psychiatric evaluation. All in all, there had been some improvement in his psychiatric state since the first psychiatric consultation on the 12th day. Haloperidol tablets, sporadically used as on demand medication to calm him, were discontinued. On the 24th day, metronidazole 500 mg administered intravenously was added to the treatment. Both antibiotics were continued through the 28th day, and then terminated.

On the 38th day the neurologist found that the electromyography (EMG) and nerve conduction studies showed changes consistent with a sensorimotor polyneuropathy affecting his lower extremities. There was no EMG pathology in his upper extremities. The EMG/neurography findings were not compatible with motor neuron disease or acute polyneuropathy. No causal explanation for his dysarthria and dysphagia was found.

The one symptom that he confirmed on all psychiatric consultations (that is, on day 12, 23, 31, 32, 35, and 42 after admission) was anxiety. This was a generalized anxiety with fluctuating intensity that responded satisfactorily to diazepam 2–2.5 mg intravenously administered 4–5 times a day. The anxiety stressed him much more when he was left alone in his room. Having a nurse or a family member nearby calmed him significantly. Apart from adding antibiotics for pneumonia, the regular medication was re-evaluated throughout the course. Antidiabetic treatment was switched from metformin tablets to insulin in order to improve his blood glucose level. The antipsychotic medication (olanzapine) was reduced to 10 mg per day as we suspected the drug to be a cause of his hypersomnia and fatigue. Despite a range of efforts from specialists in orthopedic surgery, hand surgery, anesthesiology, pulmonology, neurology, and psychiatry, our patient did not recover. He died 44 days after being admitted to hospital.

### The abnormal hand postures

**Fig. 1:**
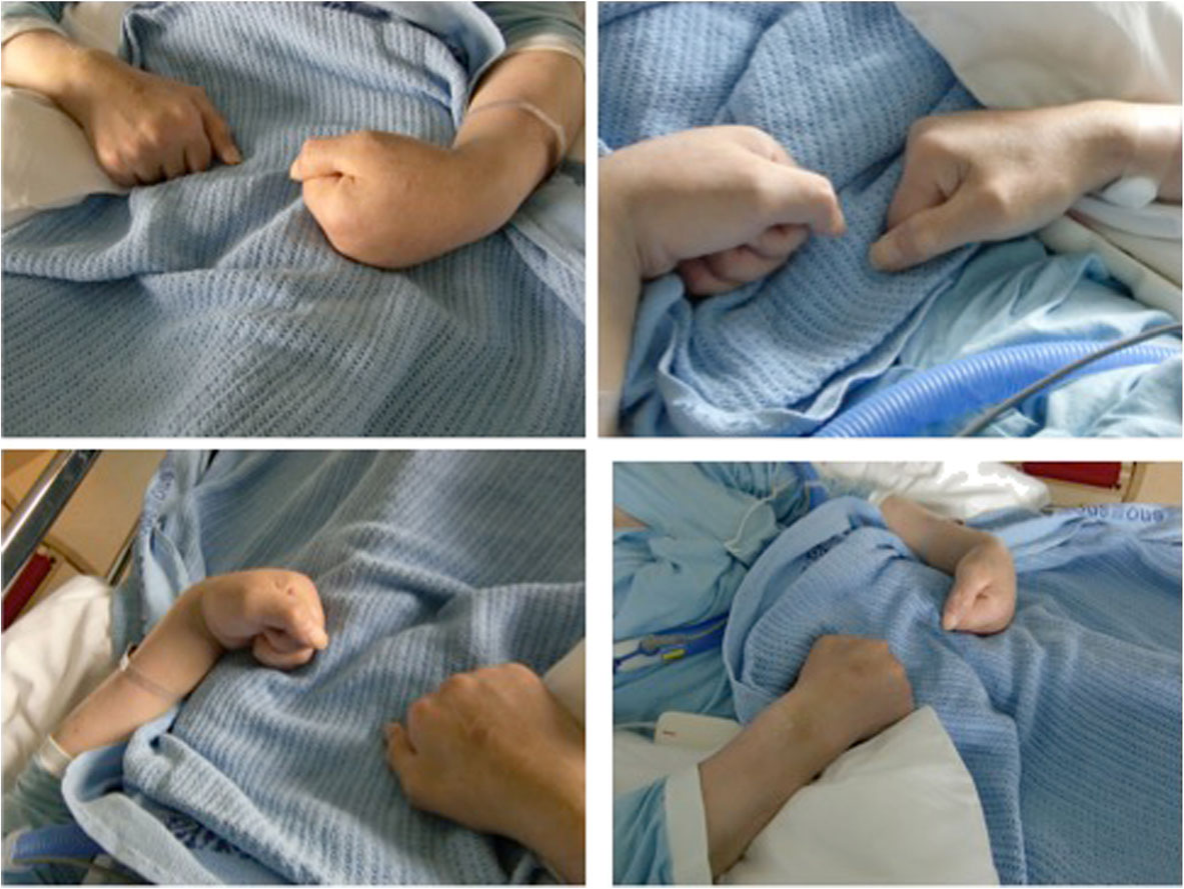
The patient’s clenched fists seen from different angles

The focus of this case presentation, however, is on the unusual observation of his clenched hands. Thus, we have to step back. During the second psychiatric consultation on day 23 after admission, he was observed clutching his hands onto the side rails of the bed. With some assistance he managed to let go of the rails, but his hands were still tightly clenched. When asked if he could extend his four ulnar fingers he only managed a slight active extension of them, just enough to let the doctor inspect and palpate his palms. On the four later psychiatric consultations, he no longer held onto the side rails. Both hands from now on lay on the duvet with his wrists in palmar flexion, the left one more strongly than the right one. His four ulnar fingers were fully flexed giving the impression of clenched fists (Fig. 1).

During these later examinations, he was still unable to open his hands voluntarily. Neither could he extend his wrists. On testing for passive extension of the wrist joints, proximal and distal interphalangeal joints, and metacarpophalangeal joints of his four ulnar fingers only slight extension was allowed for. His thumbs, however, could be fully extended, although with some resistance.

During the extension of his four ulnar fingers there was a resistance that increased proportionally to the force applied by the examiner, giving it an “elastic feel.” Furthermore, there was a non-pitting swelling on the dorsum of his left hand and lower arm. Passive extension of his fingers allowed for examinations of his palms. There was no visible or palpable sign of Dupuytren’s contracture on either side. Neither were there signs of traumas to the hands. However, he had small wounds in the left fossa cubiti caused by syringes and peripheral venous catheters associated with blood test and intravenous infusions. This could have been the culprit for the abovementioned swelling.

The neurological work-up revealed no plausible organic pathology.

Attempts to treat the clenched hands were obsolete as our patient was unable to cooperate in any way. However, he accepted a palliative application of hand orthoses that counteracted the wrist flexion to some degree during the last week of his life. According to information from family members he had had normal function of his hands prior to this hospital stay. They had a theory that his holding his hands clutched on the side rails was his attempt to prevent falling or being pulled out of his bed. Our patient himself could not explain why his hands were clenched. He had no pain in his hands, but he confirmed having more or less continuous anxiety during all six psychiatric consultations. Every attempt at mobilization in order to get him out of bed failed as he resisted both verbally, by crying out, and physically.

On day 42, a junior doctor at the Department of Hand Surgery responded to a request to examine our patient. After discussing the case with her senior colleagues, the doctor could not conclude on any plausible organic disorder. She recommended putting some insulating material between fingertips and palms to prevent maceration and wounding. She also suggested a repeat neurological examination in case he improved.

An autopsy concluded that the cause of death was aspiration pneumonia. In addition, an old infarction was found in the pons and medulla oblongata. Furthermore, there were discrete thickenings of blood vessels and old, small perivascular infarcts consistent with lacunar state in the brain. His relatives had never observed or heard our patient report symptoms compatible with stroke or cerebral insults prior to the current illness course.

## Discussion

Our patient was hospitalized after a fall that caused a left hip fracture that was operated on. Due to the various medical complications the course of his illness ended fatally after 1.5 months. Three weeks after admission a peculiar phenomenon of strongly clenched fists appeared. As there was no plausible organic cause for this, a psychiatric disorder, clenched fist syndrome (CFS), was suggested. In the literature, the nosological status of CFS is unclear. This case presentation is a first step in increasing the understanding of this syndrome from a psychiatric perspective.

The diagnostic work-up and treatment running parallel during this very sick patient’s hospital stay illustrate the complexity of clinical medicine – “medicine-at-work” – when there are several disorders with fluctuating clinical manifestations demanding collaboration by various medical specialists. Clinical medicine very often entails an inherent uncertainty regarding diagnosis, treatment, and prognosis. Although we strive for certainty, we often have to settle for temporary and tentative diagnoses. Thus we feel this report is illustrative of medicine in practice.

Our patient had been a heavy tobacco smoker for several decades and 5 years earlier was diagnosed as having diabetes mellitus type 2. Tobacco smoking and diabetes are risk factors for cerebral (and general) atherosclerosis, and it seems likely that these risk factors could have been the cause of our patient’s pathoanatomical findings at autopsy. In hindsight, the brain autopsy findings might have contributed to his dysarthric speech, dysphagia, and dizziness, maybe justifying a retrospective diagnosis of pseudobulbar palsy. The intermittent slight lead pipe rigidity of his upper left extremity might be explained either by the vascular changes or by his antipsychotic medication.

It is relevant to ask whether the cerebral lesions could have caused the clenched fists. In considering this question we first have to take into account the various relevant differential diagnoses. In order to diagnose an abnormally clenched fist as a non-organic disorder, we ought to exclude these organic disorders: (1) Dupuytren’s contracture, (2) collagen diseases, (3) rheumatic arthritis, (4) camptodactyly, (5) arthrogryposis multiplex congenita, (6) progressive systemic sclerosis, (7) eosinophilic fasciitis, (8) cerebrovascular disorders, (9) peripheral nerve injuries, (10) complex regional pain syndrome, (11) corticobasal ganglionic degeneration, and (12) late complication of Parkinson’s disease [7, 8].

The slight increase in muscle tone, described in our case as being lead pipe rigidity, was – as already mentioned – possibly caused by the antipsychotic medication. However, the clenching of his fists is not a typical extrapyramidal side effect. Our patient had no known physical disease of his hands prior to the clenching of his fists. The onset was abrupt and symmetrical. The clenching onto the bed rails was most probably motivated by his anxiety. There were no known acute cerebrovascular occurrences prior to the hands clenching. In addition, spasticity following a stroke affecting the upper motor neuron usually takes some days or weeks to develop. Initially, upper motor neuron lesions cause flaccid paresis or paralysis. The muscular contraction of our patient’s hands was strong and increased proportionally with the force applied when attempting to extend fingers and wrist joints, that is, qualitatively different from the lead pipe rigidity that was present prior to the clenching. It is hard to conceive of any of the abovementioned diseases (numbers 1–12), other than the cerebrovascular category (number 8), to start acutely and symmetrically. Besides, the abovementioned diseases (that is, numbers 1–7 and numbers 9–12), each have their distinctive clinical features that were not present. In sum, we find it reasonable to consider a psychopathological mechanism the most likely cause. However, the diagnostic work-up of the clenched fists could have been more systematic from the start and throughout the course. Obviously, the “hand problem” was of minor importance compared with all the other serious medical complications.

The conversion disorder we suggest as compatible with our case is variously labelled “CFS” [9] and “the psycho-flexed hand” [10]. This syndrome seems not to have been described in medical or psychiatric literature before E. Mitchell Hendrix and collaborators in 1978 published a case report on the treatment of “an ‘hysterically’ clenched fist” [11]. In 1980 Simmons and Vasile published a case series of five patients [9]. They labelled the phenomenon “CFS” whereas Frykman, Wood, and Miller, based on their case series 3 years later, chose a different term, “the psycho-flexed hand” [10]. The features were very similar, and it appeared obvious that it was essentially the same entity. Today, CFS seems to be the favored term.

There is a variety of psychopathologies affecting the upper extremity. These can be difficult to detect and diagnose correctly. In order to alleviate some of the confusion that can arise, researchers have proposed classifications for these disorders [7, 12, 13]. In 1991, Grunert *et al.* suggested three categories of factitious disorders: (i) factitious lymphedema, (ii) self-mutilation and wound manipulation, and (iii) finger and hand deformities [12]. They put CFS in the third factitious category, that is, finger and hand deformities, although the original publication on the syndrome [9] emphasized it being a *conversion* disorder. The two categories – factitious and conversion – are mutually exclusive. A conversion disorder cannot be a subgroup of the factitious disorders. In 2008, Mary Eldridge and collaborators, among them Grunert, published a “*Streamlined classification of psychopathological hand disorders*” [13] as an up-to-date revision of their classification from 1991. The third category, finger and hand deformities, was wisely relabeled as psychopathological dystonias, which comprised conversion disorders, factitious disorders, and malingering. In other words, the authors acknowledged conversion as a designation for at least some hand disorders.

To clarify, conversion disorders are relabeled *dissociative disorders* according to Chapter V “ICD-10 classification of mental and behavioural disorders” of the *International Statistical Classification of Diseases and Related Health Problems*, 10th version (ICD-10) [14]. If the CFS were to be given status as a formal diagnosis in the ICD-10 classification, the closest fit would be *F44.4 Dissociative motor disorder.* As Eldridge and co-authors note, this disorder is unconsciously motivated and unconsciously produced, while factitious disorders are unconsciously motivated, but consciously produced [13] (see Table 1).

**Table 1:** Differences between conversion disorders, factitious disorders, and malingering

Disorder	ICD-10 alphanumeric code	Intention/motivation	Production of symptom
Conversion	F44	Unconscious	Unconscious
Factitious	F68.1	Unconscious	Conscious
Malingering	Z76.5	Conscious	Conscious

*ICD-10The International Statistical Classification of Diseases and Related Health Problems*, 10th version

This makes good sense in theory. Alas, in clinical practice, the distinction is not always that clear. A factitious disorder cannot be diagnosed with certainty unless the patient admits to having produced the physical sign(s), or, alternatively, health personnel have observed this production. Also, it can be hard to find a clear distinction between unconscious and conscious production of symptoms or signs.

Apart from the muscular contraction of flexor muscles in CFS, other major features are [9, 10]:

- A minor physical trauma has often occurred a short time before the fists become clenched.
- A swelling of the hand may occur.
- There may be some maceration of the palm as a result of faulty hygiene.
- There is often pain with passive extension.
- There is usually no pain with passive flexion.
- There are normal findings on EMG and nerve conduction studies.
- EMG may indicate muscle contraction of finger flexors with passive extension.
- Examination performed during anesthesia often reveals a full range of motion.
- Often, only the three ulnar fingers are affected.
- The patient is often relatively unconcerned about his or her dysfunctional hands (*la belle indifférence*).
- All relevant somatic differential diagnoses have been excluded.

The diagnosis of CFS is – at the time being – typological, that is, based on its similarity to those cases that have been described in the medical literature so far. Although not very experienced with conversion disorders affecting the hand, we concluded that our patient most likely had CFS. He had the characteristic clenched hands compatible with the description of CFS. He had had a physical trauma (although not directly to his hands) a few weeks earlier when he fell on the floor. EMG and nerve conduction studies in his upper extremities were normal. All fingers were affected although his thumbs to a lesser degree. The swelling on his left hand was probably caused by repeated blood tests and intravenous infusions administered to several places on his arm. There was no indication that it was self-inflicted.

Descriptive psychiatric diagnoses that have been found to pre-date the onset of CFS are depression, schizophrenia, “obsessive-compulsive character,” and “major depression … with concurrent dependent and borderline personality disorder” [9, 10, 15]. Psychiatric/psychological evaluation beyond descriptive diagnoses can give some clues to understanding the psychodynamics. In teenagers there is usually a family conflict [3, 9]. Simmons and Vasile noted in their patients “… a consistent theme of repressed anger … The hand bound into a fist symbolically expresses anger …” [9]. The authors also pointed to one of their young patients, a 14-year-old girl having “… rigid mechanisms of avoidance and denial as defences against expressions of impulses” and “… flat rejection that emotional factors could be contributing to her disability” [9]. Patients have also been described as “… introverts, shy, timid, retiring, and unable to express anger” [10]. Grunert *et al.* described a group of 18 patients with psychopathological hand disorders of whom 11 had CFS [12]. Many of these were described as “rather passive, emotionally needy individuals who tended to focus psychological conflicts on physical symptoms.” Another group was characterized as “…angry, hostile persons who were suspicious and tended to act out their anger maladaptively.” The first group had the best prognosis with regard to returning to work after treatment.

As to the predisposing or precipitating psychological factors for our patient’s disorder, we must rely more on our own speculation than on a sound comprehensive anamnesis. Our patient had for several months been apprehensive about walking up and down staircases. This was understandable as he was dizzy and walked unsteadily. After the fall on the floor a series of events occurred that might have caused his anxiety to increase: the hospitalization, the medical work-up including the noisy and claustrophobic MRI, the hip surgery, the postoperative complications including a short delirious episode, and well-meaning health personnel trying to mobilize him against his will. From the perspective of an anxious and psychotic person with delusions, hallucinations, and speech problems, it is not hard to imagine that the hospitalization with all its alienating elements could have been a very stressful experience. This might have activated an acute stress reaction, and an equivalent to the flight, fight, or freeze-response. He could not flee. But he could “fight” to some degree, for instance by yelling out if he saw that he was about to be left alone in his room. Furthermore, maybe his hands clutching onto the side rails was part of a freeze-response. From his perspective, this is a sensible thing to do if you fear that somebody would try to pull you out of your bed, which was his relatives’ hypothesis. As he deteriorated and let go of the side rails, his hands were still clenched. The once adaptive response was no longer adaptive. Why his hands would not open, we cannot easily explain. Or, if he felt that he had had “success” with his clenched fists up until then, why give it up?

If this rather short and simple psychodynamic hypothesis has some validity, we might not have to invoke a more complex psychoanalytic explanation assuming a subconscious intrapsychic conflict resulting in the physical symptom, that is, clenched fists, as a symbolic representation of this conflict [16]. According to the “law of parsimony,” also called Occam’s razor, the most simplistic theory, that is, the one with the fewest assumptions, will often be satisfactory in practical medicine although spurious from a strictly scientific point of view [17]. Anyhow, to get a better understanding of the psychological underpinnings, we would have had to wait until he was able to communicate better. Unfortunately, he died before we reached that point.

In 2008 Batra *et al.* described the “psychoflexed hand” as “…a rarely described and poorly understood condition, which has been omitted from the latest editions of several hand surgery texts” [18]. However, the authoritative *Green’s Operative Hand Surgery,* authored by S.W. Wolfe and associates, does in fact include this disorder in its chapter on factitious disorders, published in 2011 [19]. Interestingly, neither of the two major textbooks in psychiatry mentions CFS in their most up-to-date editions [20, 21].

## Conclusions

Ours was a complex case, and there was clinical uncertainty. However, we believe that the clenching of our patient’s fists is best explained as a conversion disorder, CFS. There is still insufficient clinical data on CFS. Further research should strive to gain more knowledge on etiology, pathogenesis, clinical manifestations, and treatment. Also, the nosological status should be clarified. Should it be labelled a syndrome, a disorder, a reaction, a conversion reaction, a phenomenon, or an entity? All these designations have been used in articles about the CFS/psycho-flexed hand. In itself, that is an indication of the unresolved nosological status. The medical community will hopefully design studies that can broaden our understanding. We also urge the psychiatric community to disseminate the current knowledge, report on further observations, and collaborate with colleagues from other medical specialties that encounter this or similar disorders.

## Abbreviations

CFS: Clenched fist syndrome
EMG: Electromyography
ICD-10: *The International Statistical Classification of Diseases and Related Health Problems*, 10th version.
MRI: Magnetic resonance imaging

## References

[1] Koranyi EK. Morbidity and rate of physical illnesses in a psychiatric clinic population. Arch Gen Psychiatry. 1979;36:414–9. 10.1001/archpsyc.1979.01780040056006 426608

[2] Stone J, Smyth R, Carson A, Warlow C, Sharpe M. *La belle indifférence* in conversion symptoms and hysteria: systematic review. Br J Psychiatry. 2006;188:204–9. 10.1192/bjp.188.3.204. 16507959

[3] Nissen T, Wynn R. The recent history of the clinical case report: a narrative review. J R Soc Med Sh Rep. 2012;3:87. 10.1258/shorts.2012.012046. PMC3545337 23476729

[4] Nissen T, Wynn R. The clinical case report: a review of its merits and limitations. BMC Res Notes. 2014;7:264. 10.1186/1756-0500-7-264. PMC4001358 24758689

[5] Nissen T, Wynn R. The history of the case report: a selective review. JRSM Open. 2014;5(4):1–5. 10.1177/2054270414523410. PMC4012665 25057387

[6] Gagnier JJ, Kienle G, Altman DG, Moher D, Sox H, Riley D, The CARE group. The CARE guidelines: consensus-based clinical case reporting guideline development. J Med Case Rep. 2013;7(1):223. 10.1186/1752-1947-7-223 PMC3844611 24228906

[7] Petrella L, Belkheyar Z, Oberlin C. The psychoflexed hand: new perspectives in diagnosis, classification and treatment. Chir Main. 2013;32:245–50. 10.1016/j.main.2013.04.005 23712089

[8] Cordivari C, Misra P, Catania S, Lees AJ. Treatment of dystonic clenched fist with botulinum toxin. Mov Disord. 2001;16:907–13. 10.1002/mds.1186 11746621

[9] Simmons BP, Vasile RG. The clenched fist syndrome. J Hand Surg. 1980;5:420–7. 10.1016/s0363-5023(80)80071-2 7430578

[10] Frykman GK, Wood VE, Miller EB. The psycho-flexed hand. Clin Orthop Relat Res. 1983;174:153–7. 6831798

[11] Mitchell Hendrix E, Thompson LM, Rau BW. Behavioral treatment of an “hysterically” clenched fist. J Behav Exp Psychiat. 1978;9:273–6.

[12] Grunert BK, Sanger JR, Matloub HS, Yousif NJ. Classifications system for factitious syndromes in the hand with implications for treatment. J Hand Surg Am. 1991;16A:1027–30. 10.1016/s0363-5023(10)80062-0 1748746

[13] Eldridge M, Grunert BK, Matloub HS. Streamlined classification of psychopathological hand disorders: a literature review. Hand (N Y). 2008;3:118–28. 10.1007/s11552-007-9072-0. PMC2529133 18780087

[14] World Health Organization. The ICD-10 classification of mental and behavioural disorders: clinical descriptions and diagnostic guidelines. Geneva: WHO; 1992.

[15] Cuénod Ph SD, Degive C, Della Santa DR. Psychogenic spastic hand. Ann Hand Surg. 1996;15:100–8. 10.1016/S0753-9053(96)80015-9. 8816092

[16] Gabbard GO. Psychodynamic psychiatry in clinical practice 3rd ed. Washington: American Psychiatric Press; 2000.

[17] Hillard AA, Weinberger SE, Tierney LM Jr, Midthun DE, Saint S. Clinical problem-solving. Occam’s razor versus Saint’s triad. N Engl J Med. 2004;350:599–603. 10.1056/NEJMcps031794. 14762188

[18] Batra S, Sarasin A, Gul A, Kanvinde R. Psychoflexed hand: a forgotten entity. A case report and review of the literature. Int J Clin Pract. 2008;62:1634–6. 10.1111/j.1742-1241.2007.01086.x. 18822035

[19] Louis DS, Kasdan ML. Factitious disorders. In: Wolfe SW, Hotchkiss RN, Pederson WC, Kozin SH, editors. Green’s operative hand surgery. 6th ed. Philadelphia: Elsevier; 2011. p. 1989–92.

[20] Gelder MG, Andreasen NC, López-Ibor JJ Jr, Geddes JR. New Oxford textbook of psychiatry. 2nd ed. Oxford: Oxford University Press; 2009.

[21] Sadock BJ, Sadock VA, Ruiz PR. Kaplan & Sadock’s comprehensive textbook of psychiatry. 10th ed. Philadelphia: Wolters Kluver; 2017.

